# Diosmectite inhibits the interaction between SARS-CoV-2 and human enterocytes by trapping viral particles, thereby preventing NF-kappaB activation and CXCL10 secretion

**DOI:** 10.1038/s41598-021-01217-2

**Published:** 2021-11-05

**Authors:** Marco Poeta, Valentina Cioffi, Vittoria Buccigrossi, Merlin Nanayakkara, Melissa Baggieri, Roberto Peltrini, Angela Amoresano, Fabio Magurano, Alfredo Guarino

**Affiliations:** 1 Pediatrics Division, Department of Translational Medical Science, University of Naples Federico II, 80138 Naples, Italy; 2 Department of Infectious Diseases, National Institute of Health, Rome, Italy; 3 Department of Public Health, University of Naples Federico II, Naples, Italy; 4 Department of Chemical Sciences, University of Naples Federico II, Naples, Italy

**Keywords:** Gastrointestinal models, Gastrointestinal system, Public health, Gastroenterology, Pathogenesis

## Abstract

SARS-CoV-2 enters the intestine by the spike protein binding to angiotensin-converting enzyme 2 (ACE2) receptors in enterocyte apical membranes, leading to diarrhea in some patients. Early treatment of COVID-19-associated diarrhea could relieve symptoms and limit viral spread within the gastrointestinal (GI) tract. Diosmectite, an aluminomagnesium silicate adsorbent clay with antidiarrheal effects, is recommended in some COVID-19 management protocols. In rotavirus models, diosmectite prevents pathogenic effects by binding the virus and its enterotoxin. We tested the trapping and anti-inflammatory properties of diosmectite in a SARS-CoV-2 model. Trapping effects were tested in Caco-2 cells using spike protein receptor-binding domain (RBD) and heat-inactivated SARS-CoV-2 preparations. Trapping was assessed by immunofluorescence, alone or in the presence of cells. The effect of diosmectite on nuclear factor kappa B (NF-kappaB) activation and CXCL10 secretion induced by the spike protein RBD and heat-inactivated SARS-CoV-2 were analyzed by Western blot and ELISA, respectively. Diosmectite bound the spike protein RBD and SARS-CoV-2 preparation, and inhibited interaction of the spike protein RBD with ACE2 receptors on the Caco-2 cell surface. Diosmectite exposure also inhibited NF-kappaB activation and CXCL10 secretion. These data provide direct evidence that diosmectite can bind SARS-CoV-2 components and inhibit downstream inflammation, supporting a mechanistic rationale for consideration of diosmectite as a management option for COVID-19-associated diarrhea.

## Introduction

A substantial number of patients with the novel coronavirus disease 2019 (COVID-19) experience gastrointestinal (GI) symptoms^1–3^. Prevalence estimates vary between studies, with higher rates noted among children^4^, but up to 39.6% of all patients with COVID-19 are believed to experience associated diarrhea^5^. The prognostic implications of diarrhea in patients with COVID-19 requires further investigation, but there is increasing evidence of correlations with both disease severity and survival^6,7^.

Severe acute respiratory syndrome coronavirus 2 (SARS-CoV-2) gains entry to host cells by exploiting the angiotensin-converting enzyme 2 (ACE2) receptor, which is highly expressed in absorptive enterocytes from the ileum and colon^8^. ACE2 expression in the GI tract is thought to lead to dysregulation of intestinal ACE2, affecting the expression of antimicrobial peptides and homeostasis of the gut microbiome^6^. Infection of intestinal cells with SARS-CoV-2 also activates several signaling pathways (e.g., nuclear factor kappa B [NF-kappaB] and TGF beta, directly; PI3K/AKT, Ras/Raf/MAPK/STAT and Src via the activation of EGFR) causing further, indirect, pathogenic effects^9^. In addition, there is evidence of SARS-CoV-2 localization in the cytoplasm of gastric, duodenal, and rectal glandular epithelial cells^10^, and data suggest that fecal–oral transmission of SARS-CoV-2 may be a route of infection^11^, highlighting the importance of GI management considerations in patients with COVID-19.

The development of diarrhea in patients with COVID-19 may naturally prompt the use of antidiarrheal agents, but caution has been recommended with respect to the use of agents that slow intestinal motility^12^. These agents extend transit time and may delay the clearance of the SARS-CoV-2 pathogen from the gut^12,13^. Thus, antimotility drugs have the potential to prolong the course of SARS-CoV-2 infection and increase the risk of a more severe COVID-19 disease course^12^. Based on this theoretical rationale, it has been highlighted that it may be relevant to consider use of antidiarrheal agents with a mode of action that does not delay intestinal transit time^12^. Relevant drug classes in patients with diarrhea include antisecretory agents with inhibitory actions on enkephalinase and adsorbents that have the potential to bind digestive mucus and toxins, as well as reduce water loss^12^. Indeed, recommendations for the use of the adsorbent dioctahedral smectite (diosmectite) have appeared in a number of local protocols and national guidelines for the management of GI symptoms in patients with COVID-19^14–16^.

Diosmectite is a well-defined multilamellar argillaceous compound recommended for the management of several GI diseases in adults and children^17,18^. At the time of writing, there are no empirical data on the efficacy of diosmectite in patients with COVID-19, thus clinical recommendations for its use in these patients are based on an inferred clinical rationale.

Diosmectite is a natural aluminomagnesium silicate adsorbent clay that exerts antidiarrheal effects on the intestinal epithelium^18–20^. Its effects are localized in the intestinal lumen or luminal surface of the epithelium where it adsorbs toxins, bacteria, and virus complexes^17^. There is also evidence that it can counteract rotavirus-induced cytotoxic damage and ion secretion via inhibition of viral replication and expression of the non-structural viral protein NSP4^19,21^. Before the emergence of COVID-19, an in vitro evaluation of several adsorbent clays (including a range of smectites) found a high adsorption capability for coronavirus, with high affinity (0.06–3.09% desorption). The study authors noted, however, that adsorbent-bound virus complexes retained infectivity^13^, lending further support to the rationale for avoiding diarrheal management approaches that delay intestinal transit time.

To evaluate the theoretical rationale for the use of diosmectite for the management of diarrhea in patients with COVID-19, we conducted in vitro trapping experiments to assess the ability of diosmectite to bind to SARS-CoV-2 components and inhibit downstream inflammation.

## Results

The protein concentration of the heat-treated SARS-CoV-2 preparation was 523 μg/mL; it contained 26 μg/mL of spike protein receptor-binding domain (RBD) (i.e., ~ 5% of total protein).

### Diosmectite trapping capability

**Figure 1:**
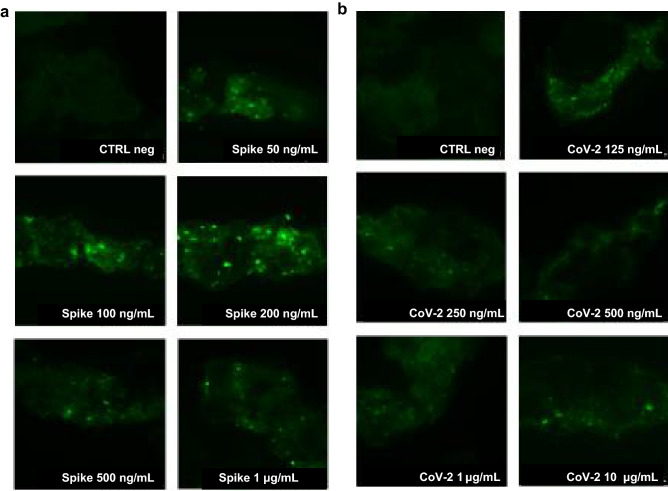
Trapping of viral particles by diosmectite. Diosmectite was incubated with spike protein RBD (**a**) and heat-inactivated severe acute respiratory syndrome coronavirus 2 (CoV-2) (**b**) at different concentrations. The suspension was then probed with the anti-spike protein RBD antibody. Diosmectite alone was used as a negative control (CTRL). Images are at × 1000 magnification and represent three separate experiments.

After 1 h of incubation, diosmectite bound the spike protein RBD, based on the presence of specific fluorescence. There was no signal with diosmectite alone, which served as a negative control. Fluorescence intensity was consistent across all samples evaluated, with intensity positively correlated with spike protein RBD concentration (Fig. 1a).

The trapping capability of diosmectite was also observed when incubated with the SARS-CoV-2 preparation; again, the fluorescence (green signal) intensity was dependent of the protein concentration of the viral preparation (Fig. 1b).

**Figure 2:**
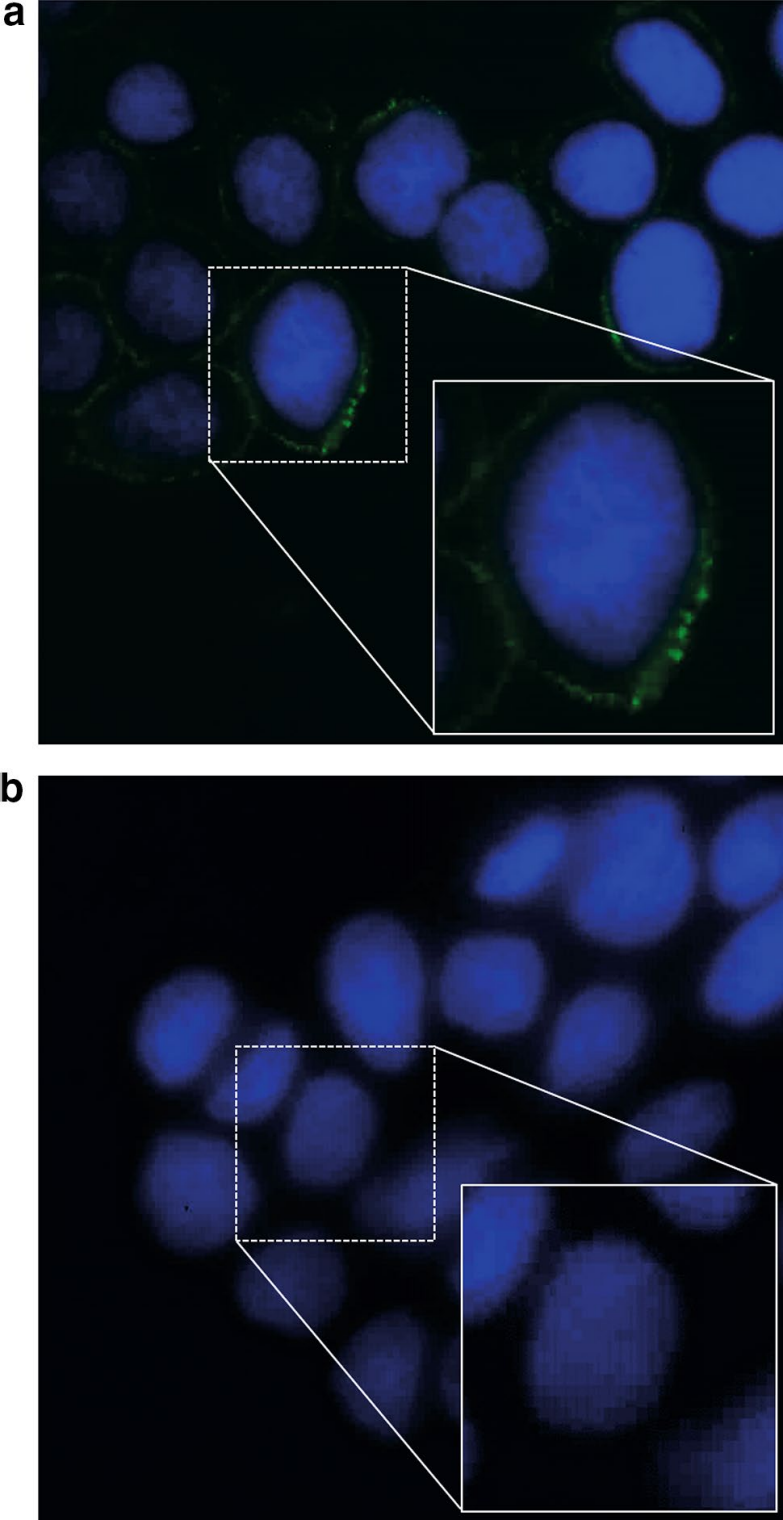
Spike-adhesion to the Caco-2 cell surface in the absence **(a)** or presence **(b)** of diosmectite, as shown by immunofluorescence microscopy. Spike protein RBD is shown after being probed with the primary and secondary fluorescein isothiocyanate-conjugated antibody (green). Nuclei were stained using Hoechst (blue). Images shown are × 1000 magnification. A detail of a single cell is shown in the square. (**a**) Caco-2 cells incubated with spike protein RBD. (**b**) Caco-2 cells pre-incubated with diosmectite and co-incubated with spike protein RBD.

Immunofluorescence microscopy showed that the spike protein RBD interacts with the surface of Caco-2 cells in the absence of diosmectite after 1 h of incubation (Fig. 2a). When cells were pre-treated with diosmectite (100 mg/mL) for 15 min, enterocyte upload of the spike protein RBD was dramatically reduced, shown by the absence of green florescence (Fig. 2b).

### Effects on NF-kappaB activation

**Figure 3:**
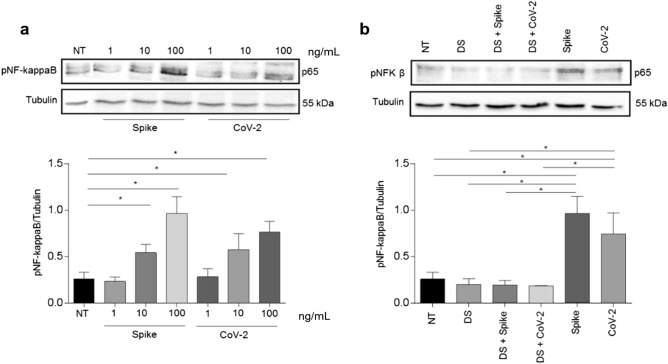
NF-kappaB activation and preventive effect of diosmectite. (**a**) Western blot analysis of protein lysates from Caco-2 cells treated for 24 h with different concentrations of spike protein RBD (Spike) and heat-inactivated SARS-CoV-2 (CoV-2) as indicated. (**b**) Western blot analysis of protein lysates from Caco-2 cells treated with spike protein RBD (100 ng/mL) and heat-inactivated CoV-2 (100 ng/mL) for 24 h alone and after pretreatment with diosmectite (DS). The upper line was blotted with anti-pNF-kappaB antibodies and the lower line was blotted with anti-tubulin antibodies as a loading control. A representative image from three independent experiments is shown. The relative levels of pNF-kappaB were normalized to tubulin levels. Bars indicate the means and lines the standard deviations of the three independent experiments. Student’s t-test. *p ≤ 0.05. *SARS-CoV-2* severe acute respiratory syndrome coronavirus 2, *DS* diosmectite, *NF-kappaB* nuclear factor kappaB, *NT* not treated, *p65* primary antibody for NF-kappaB, *pNF-kappaB* phosphorylated NF-kappaB. Full-length blots/gels are presented in Supplementary Fig. S3.

An antibody against the phosphorylated form of NF-kappaB (pNF-kappaB) was used to evaluate the activation effect of 24-h exposure to different concentrations of spike protein RBD and the heat-inactivated SARS-CoV-2 preparation on NF-kappaB in Caco-2 cells (Fig. 3a). For both spike protein RBD and heat-inactivated SARS-CoV-2, the most effective concentration was 100 ng/mL. Diosmectite prevented activation of NF-kappaB induced by both spike protein RBD and heat-inactivated SARS-CoV-2 preparations (Fig. 3b). Similar results were obtained after a shorter exposure period (1 h) (Supplementary Fig. S1). Diosmectite alone did not induce any change in NF-kappaB.

### Effects on interferon-γ (IFN-γ) and CXCL10 secretion

Supernatants of Caco-2 cell cultures were collected 24 h after stimulation with spike protein RBD and the heat-inactivated SARS-CoV-2 preparation. The highest dose of spike protein RBD (100 ng/mL) induced a statistically significant (twofold) increase in IFN-γ compared with control cells. The highest concentration of heat-inactivated SARS-CoV-2 preparation (100 ng/mL) also induced an increase in IFN-γ, although the increase was not statistically significant. Lower doses of both preparations had no effect on IFN-γ secretion (Supplementary Fig. S2).

**Figure 4:**
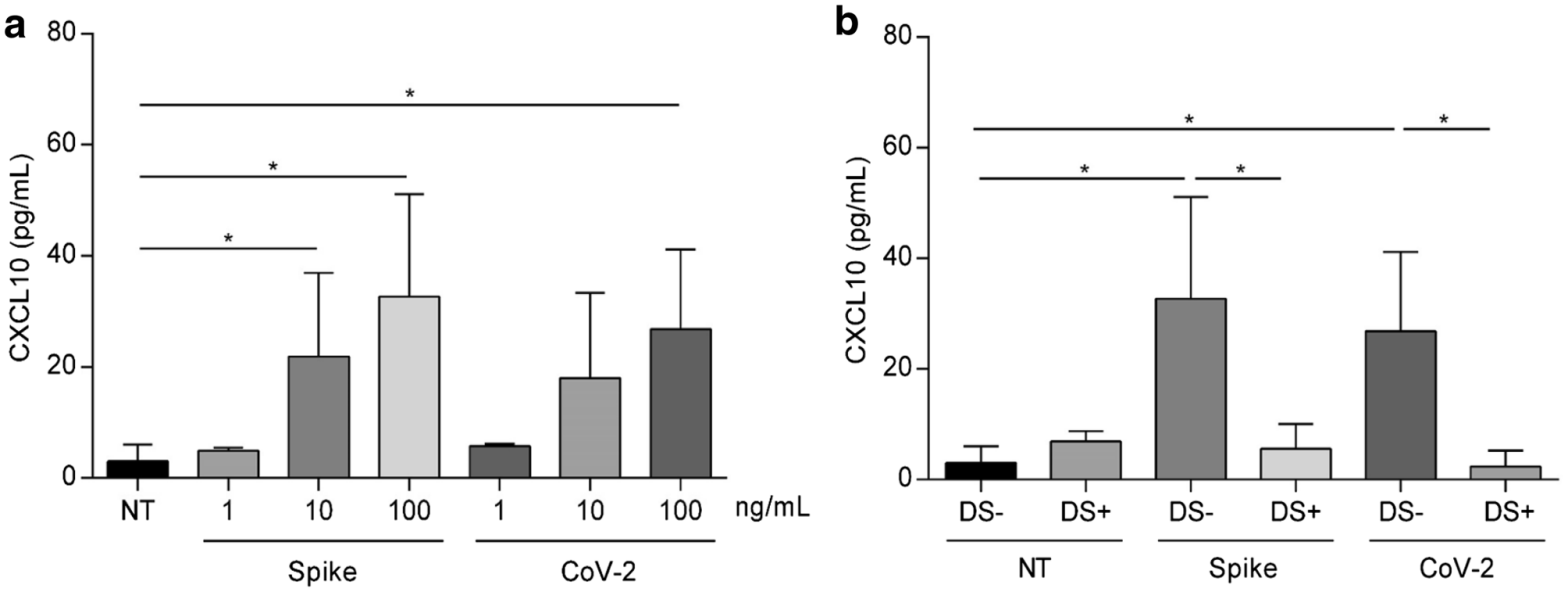
CXCL10 secretion and preventive effect of diosmectite. (**a**) Levels of CXCL10 measured by ELISA in supernatants of Caco-2 cell cultures treated for 24 h with different concentrations of spike protein RBD (Spike) and heat-inactivated SARS-CoV-2 (CoV-2) as indicated. (**b**) Levels of CXCL10 measured by ELISA in supernatants of Caco-2 cell cultures treated for 24 h with spike protein RBD (100 ng/mL) and heat-inactivated SARS-CoV-2 (100 ng/mL) alone and after pretreatment with diosmectite (DS). Bars indicate the means and lines the standard deviations of three independent experiments. Student’s t‑test. *p ≤ 0.05. *ELISA* enzyme-linked immunosorbent assay, *DS* diosmectite, *NT* not treated, *SARS-CoV-2* severe acute respiratory syndrome coronavirus 2.

Both spike protein RBD and heat-inactivated SARS-CoV-2 preparation induced the secretion of the chemokine CXCL10 in Caco-2 cells in a dose-dependent manner; the highest response was achieved at a concentration of 100 ng/mL for both preparations (ninefold increase for spike protein RBD compared with control cells) (Fig. 4a).

The most effective concentrations of spike protein RBD and heat-inactivated SARS-CoV-2 (100 ng/mL for both) on CXCL10 were subsequently used to test the effects of diosmectite. In the presence of diosmectite the induction of CXCL10 was inhibited (Fig. 4b). Diosmectite alone did not induce any change in CXCL10 levels.

## Discussion

The SARS-CoV-2 spike proteins bind to ACE2 receptors located on the brush border of enterocytes and exert direct pathogenic effects within the intestinal tract. The direct viral effects of cell invasion are further exacerbated by indirect inflammatory effects and disruption of the intestinal microbiota^22^.

Our model provides novel, direct evidence of trapping of SARS-CoV-2 spike proteins within the diosmectite clay structure. Diosmectite has been shown to reduce diarrhea in children with acute gastroenteritis^23^ and, based on compelling efficacy data, is endorsed by international guidelines for the management of acute pediatric gastroenteritis^18^. The mechanism of action of diosmectite against rotavirus (the leading cause of severe gastroenteritis in children) relates to its ability to adsorb viral components^18,21^. SARS-CoV-2 frequently induces GI symptoms^6,24^ and family outbreaks may be associated with prolonged fecal viral shedding^11^. Our data suggest that diosmectite can bind heat-inactivated SARS-Cov-2 and spike protein RBD, thereby preventing the potential interaction with ACE2 receptors on cell surfaces; similar to the mechanism of action of diosmectite against rotavirus. These results exploit a strategy of intermolecular interference that has been observed with human defensin 5 which blocks the spike protein by cloaking ACE2^25^.

In recognition of the ability of SARS-CoV-2 to induce an inflammatory response following infection of intestinal cells, we used Caco-2 cells to test the ability of spike protein RBD and heat-inactivated SARS-CoV-2 to activate NF-kappaB and induce secretion of IFN-γ and CXCL10. Both viral components activated NF-kappaB and induced the expression of the pro-inflammatory chemokine CXCL10. An increase in CXCL10 has previously been reported in Calu-3 lung epithelial cells in mice lungs following SARS-CoV-2 infection^26^ and in COVID-19 patients^27^, implicating this chemokine as a key contributor to SARS-CoV-2-related cytokine storm. In our experimental model the IFN-γ response was of limited magnitude; however, in an in vitro study, Yeruva et al*.* demonstrated that induction of CXCL10 may be dependent on other cytokines (such as interleuchin-1β, TNF-α) either acting alone or synergistically with IFN-γ^28^. A similar scenario may occur in cases of SARS-CoV-2 intestinal infection in which the induction of CXCL10 by the spike protein may be totally or partially independent of IFN-γ, as has been observed for macrophages^29^.

There is increasing evidence that the NF-kappaB pathway is a central signaling pathway for the SARS-CoV-2 infection-induced pro-inflammatory cytokine/chemokine response, and that inhibition of NF-kappaB has the potential to inhibit both virus- and LPS-induced cytokine storm^30^. Diosmectite was able to prevent both NF-kappaB activation and CXCL10 secretion because of its ability to trap viral particles, thereby inhibiting their interaction with the cell surface and the activation of intracellular inflammatory pathways^31^. In addition to its ability to adsorb luminal antigens, diosmectite may mitigate diarrhea and contribute to reduced inflammation by increasing colonic mucin levels and via direct modulatory effects of cytokine production, as previously described in an experimental rat model of hapten-induced colitis^31^.

Diosmectite is widely used as an antidiarrheal agent in clinical settings, but no direct assessment of its activity against SARS-CoV-2 had previously been conducted. The SARS-CoV-2 trapping and anti-inflammatory effects of diosmectite demonstrated by our model, provides a theoretical rationale for the efficacy of diosmectite for the management of patients with COVID-19-associated diarrhea.

The main limitation of our study is the use of an in vitro model of human intestinal (Caco-2) cells, without gut sub-mucosa, immune cells and vascular components, which may play an additional role in intestinal inflammation. Further animal and ex vivo studies and clinical trials are warranted to provide a more comprehensive understanding of the role of the intestine in the inflammatory response to COVID-19 and SARS-CoV-2-associated diarrhea, and potential implications for patient management.

## Materials and methods

### Cell culture, virus and reagents

Caco-2 cells (Zooprophylactic institute, Brescia, Italy) derived from a colon carcinoma were used for the trapping experiments because of their previous use in COVID-19 associated research and their ability to differentiate into enterocytes of the upper villus, forming monolayers featuring ACE2 receptors^32^.

The cells were grown in high glucose (4.5 g/L) DMEM (Gibco, Thermo Fisher Scientific, Oxfordshire, UK) supplemented with fetal calf serum (FBS) (10%; Gibco), non-essential amino acids (1%), penicillin (50 mU/mL), and streptomycin (50 mg/mL).

A serum-free viral preparation of SARS-CoV-2 was obtained from the National Institute of Health (ISS, Rome, Italy)^33^. SARS-CoV-2 virus was amplified in Vero C1008 (Vero E6) cell culture. Inoculation of Vero E6 cells with SARS-CoV-2 was carried out directly in serum-free DMEM. Medium harvested from infected cells 4 days after inoculation was clarified by centrifugation and inactivated by temperature. Virus propagation and manipulation occurred in a BSL-3 laboratory setting at ISS.

The heat-inactivated preparation of SARS-CoV-2 and a commercial spike protein (RBD [V367F, SPD-S52H4] ACRO Biosystems, Newark, DE, USA) was tested in the absence or presence of diosmectite (Ipsen Consumer HealthCare, France).

Liquid chromatography-mass spectrometry was used to confirm the total protein and spike protein concentrations in the viral preparations.

### Trapping experiments

The ability of diosmectite (Ipsen Consumer HealthCare, France) to bind SARS-CoV-2 components was tested through in vitro trapping experiments without cells. Diosmectite (100 mg/mL) was directly incubated with either the heat-inactivated SARS-CoV-2 preparation or pure spike protein RBD at different concentrations (50, 100, 200, 500 and 1000 ng/mL and 0.125, 0.25, 0.50, 1 and 10 μg/mL, respectively) for 1 h at 37 °C. After centrifugation, the preparations were washed twice with PBS and then probed with the primary antibody (anti-SARS-CoV-2 RBD neutralizing antibody, human IgG [SAD-S35], ACRO Biosystems; 1:1000 dilution) for 1 h at room temperature, followed by the secondary antibody (Fluorescein isothiocyanate [FITC] conjugated polyclonal rabbit anti-Human IgG [F02020-2], Dako; 1:30 dilution) for 1 h at room temperature in a dark chamber. After incubation, the preparations were washed with PBS and left to precipitate for 1 h in a dark chamber. Supernatants were discharged and precipitates were evaluated using a Nikon Eclipse 80i epifluorescence microscope (FITC filter). The images were analyzed using the NIS-Elements D imaging software (Ver3.22.00).

### Inhibition effect of spike binding to cell surface

Cells were seeded onto 12-well plates with sterile glass slides at a density of 2 × 10^5^ cells per well, and pre-incubated with diosmectite (100 mg/mL) for 15 min at 37 °C, followed by co-incubation with spike protein RBD (8 µg/mL) for 1 h at 4 °C. The primary anti-spike protein RBD antibody (anti-SARS-CoV-2 RBD neutralizing antibody, human IgG1 [SAD-S35] ACRO Biosystems; 1:100) and the secondary FITC conjugated antibody (polyclonal rabbit anti-human IgG/FITC [F020202-2], Dako; 1:100) were used to stain spike protein RBD. Nuclei were stained with Hoechst 33342 (1 µg/mL). Cells were evaluated using a Nikon Eclipse 80i epifluorescence microscope (FITC filter). The images were analyzed using NIS-Elements D imaging software.

### Western blot

Caco-2 cells were stimulated with different concentrations of spike protein RBD and heat-inactivated SARS-CoV-2 preparation for 1 h and for 24 h at 37 °C with or without pre-treatment with diosmectite (100 mg/mL). Cells were washed twice with cold PBS and suspended in lysis buffer (Tris–HCl [50 mM, pH 7.4], EDTA [1 mM], EGTA [1 mM], MgCl_2_ [5 mM], NaCl [150 mM], Triton [1%], PMSF [1 mM], VO_4_[1 mM], aprotinin [100 × ; Sigma, Milan, Italy], and LAP [50 ×; Roche, Milan, Italy]) before incubation for 30 min on ice. Lysates were clarified by centrifugation at 16,000×*g* for 30 min at 4 °C. The obtained supernatants represented the total cell extracts. Protein content was determined using the Bio-Rad reagent protein (BIO-RAD Laboratories, Milan, Italy).

For Western blot analyses, 40 µg of total proteins were separated by SDS (10%)–polyacrylamide gel electrophoresis and transferred onto trans blot turbo (BIO-RAD). The membranes were then blocked with non-fat dry milk solution (5%) and probed with rabbit anti-phosphorylated NF-kappaB (anti-pNF-kappaB) (Elabscience, Houston, Texas, USA) and with mouse anti-tubulin (Sigma, Milan, Italy). Bands were visualized with enhanced chemiluminescence (Elabscience) using 2- to 10-min exposures. Band intensity was evaluated by integrating all of the pixels of the band without the background to calculate the average number of pixels surrounding the band^34^.

### Enzyme-linked immunosorbent assays (ELISA)

Caco-2 cells (1 × 10^5^ per well) were seeded into 24-well plates and grown until reaching 80% confluence. To study the effects on IFN-γ and CXCL10 induction, supernatants of Caco-2 cells were collected 24 h after stimulation with different concentrations of spike protein RBD and heat-inactivated SARS-CoV-2 preparation. Levels of IFN-γ and CXCL10 were measured in the supernatants by enzyme-linked immunosorbent assays (ELISA) according to the manufacturer’s protocol (Elabscience). The CXCL10 assay was repeated after 1 h pre-treatment at 37 °C of spike protein RBD and heat-inactivated SARS-CoV-2 with diosmectite (100 mg/mL).

### Statistical analysis

Comparison of NF-kappaB activity, IFN-γ and CXCL10 levels between groups was performed by Student’s t-test. Statistical significance was defined as a *p* value less than 0.05.

## Supplementary Information

Supplementary Information.

## Data Availability

Study documents, such as the study protocol and clinical study report, are not always available. Proposals should be submitted to DataSharing@Ipsen.com and will be assessed by a scientific review board. Data are available beginning 6 months and ending 5 years after publication; after this time, only raw data may be available.

## References

[1] Luo S, Zhang X, Xu H (2020). Don't overlook digestive symptoms in patient with 2019 novel coronavirus disease (COVID-19). Clin. Gastroenterol. Hepatol..

[2] Redd WD (2020). Prevalence and characteristics of gastrointestinal symptoms in patients with severe acute respiratory syndrome coronaviarus 2 infetion in the United States: A multicenter cohort study. Gastroenterology.

[3] Pan L (2020). Clinical characteristics of COVID-19 patients with digestive symptoms in Hubei, China: A descriptive, cross-sectional multicenter study. Am. J. Gastroenterol..

[4] Garazzino S (2021). Epidemiology, clinical features and prognostic factors of pediatric SARS-CoV-2 infection: Results from an Italian multicenter study. Front. Pediatr..

[5] Zhang JJ (2020). Clinical characteristics of 140 patients infected with SARS-CoV-2 in Wuhan, China. Allergy.

[6] D'Amico F, Baumgart DC, Danese S, Peyrin-Biroulet L (2020). Diarrhea during COVID-19 infection: Pathogenesis, epidemiology, prevention, and management. Clin. Gastroenterol. Hepatol..

[7] Han J (2020). Clinical and CT imaging features of SARS-CoV-2 patients presented with diarrhea. J. Infect..

[8] Barbosa da Luz B (2020). An overview of the gut side of the SARS-CoV-2 infection. Intest. Res..

[9] Hemmat N (2021). The roles of signaling pathways in SARS-CoV-2 infection; lessons learned from SARS-CoV and MERS-CoV. Arch. Virol..

[10] Lamers MM (2020). SARS-CoV-2 productively infects human gut enterocytes. Science.

[11] Yeo C, Kaushal S, Yeo D (2020). Enteric involvement of coronaviruses: Is faecal-oral transmission of SARS-CoV-2 possible?. Lancet Gastroenterol. Hepatol..

[12] Kow CS, Hasan SS (2021). The use of antimotility drugs in COVID-19 associated diarrhea. J. Infect..

[13] Clark KJ, Sarr AB, Grant PG, Phillips TD, Woode GN (1998). In vitro studies on the use of clay, clay minerals and charcoal to adsorb bovine rotavirus and bovine coronavirus. Vet. Microbiol..

[14] Mao R (2020). Expert consensus on diagnosis and treatment of COVID-19 digestive system. Chin. J. Nat. Med..

[15] Lombardy Ministry for Health. *Acts of Guidance for Integrated Hospital-Territory Management for Assistance to COVID-19 or Suspected Patients. Nov. 19, 2020)*. https://www.regione.lombardia.it/wps/portal/istituzionale/HP/istituzione/Giunta/sedute-delibere-giunta-regionale/DettaglioDelibere/delibera-3876-legislatura-11 (Accessed May 25, 2021).

[16] Ministry of Health of the Czech Republic. *Diarrhea Manual of Care for Patients with COVID-19 Hospitalized in a Standard Ward. June 25, 2020*. https://www.infekce.cz/Covid2019/ManualPece1120p.pdf (Accessed May 25, 2021).

[17] Mahraoui L, Heyman M, Plique O, Droy-Lefaix MT, Desjeux JF (1997). Apical effect of diosmectite on damage to the intestinal barrier induced by basal tumour necrosis factor-alpha. Gut.

[18] Guarino A (2014). European Society for Pediatric Gastroenterology, Hepatology, and Nutrition/European Society for Pediatric Infectious Diseases evidence-based guidelines for the management of acute gastroenteritis in children in Europe: Update 2014. J. Pediatr. Gastroenterol. Nutr..

[19] Dupont C, Vernisse B (2009). Anti-diarrheal effects of diosmectite in the treatment of acute diarrhea in children: A review. Paediatr. Drugs.

[20] World Health Organization United Nations Children's Fund (2004). WHO/UNICEF JOINT Statement: Clinical Management of Acute Diarrhea.

[21] Buccigrossi V, Russo C, Guarino A, de Freitas MB, Guarino A (2017). Mechanisms of antidiarrhoeal effects by diosmectite in human intestinal cells. Gut Pathog..

[22] Devaux CA, Lagier JC, Raoult D (2021). New insights into the physiopahtology of COVID-19: SARS-CoV-2-associated gastrointestinal illness. Front. Med..

[23] Guarino A (2001). Smectite in the treatment of acute diarrhea: A nationwide randomized controlled study of the Italian Society of Pediatric Gastroenterology and Hepatology (SIGEP) in collaboration with primary care pediatricians. SIGEP Study Group for Smectite in Acute Diarrhea. J. Pediatr. Gastroenterol. Nutr..

[24] Cheung KS (2020). Gastrointestinal manifestations of SARS-CoV-2 infection and virus load in fecal samples from a Hong Kong cohort: Systematic review and meta-analysis. Gastroenterology.

[25] Wang C (2020). Human intestinal defensin 5 inhibits SARS-CoV-2 invasion by cloaking ACE2. Gastroenterology.

[26] Callahan V (2021). The pro-inflammatory chemokines CXCL9, CXCL10 and CXCL11 are upregulated following SARS-CoV-2 infection in an AKT-dependent manner. Viruses.

[27] Tripathy AS (2021). Pro-inflammatory CXCL-10, TNF-α, IL-1β, and IL-6: Biomarkers of SARS-CoV-2 infection. Arch. Virol..

[28] Yeruva S, Ramadori G, Raddatz D (2008). NF-kappaB-dependent synergistic regulation of CXCL10 gene expression by IL-1beta and IFN-gamma in human intestinal epithelial cell lines. Int. J. Colorectal Dis..

[29] Chiok K, Hutchison K, Miller LG, Bose S, Miura TA (2021). Proinflammatory responses in SARS-CoV-2 infected and soluble spike glycoprotein S1 subunit activated human macrophages. bioRxiv.

[30] Kircheis R (2020). NF-κB pathway as a potential target for treatment of critical stage COVID-19 patients. Front. Immunol..

[31] González R (2004). Anti-inflammatory effect of diosmectite in hapten-induced colitis in the rat. Br. J. Pharmacol..

[32] Kumar S (2021). Clinically relevant cell culture models and their significance in isolation, pathogenesis, vaccine development, repurposing and screening of new drugs for SARS-CoV-2: A systematic review. Tissue Cell..

[33] Magurano F, Baggieri M, Marchi A, Rezza G, Nicoletti L (2021). SARS-CoV-2 infection: The environmental endurance of the virus can be influenced by the increase of temperature. Clin. Microbiol. Infect..

[34] Lania G (2019). Constitutive alterations in vesicular trafficking increase the sensitivity of cells from celiac disease patients to gliadin. Commun. Biol..

