# Supplementary material for: Diosmectite inhibits the interaction between SARS-CoV-2 and human enterocytes by trapping viral particles, thereby preventing NF-kappaB activation and CXCL10 secretion
Source: Sci Rep. 2021 Nov 5;11:21725. doi: 10.1038/s41598-021-01217-2 (PMC8571314; doi:10.1038/s41598-021-01217-2)
Supplement: Supplementary file 1 — Supplementary Information. [file 41598_2021_1217_MOESM1_ESM.docx]

**Supplementary Information file**

**Diosmectite inhibits the interaction between SARS-CoV-2 and human enterocytes by trapping viral particles, thereby preventing NF‑kappaB activation and CXCL10 secretion**

Marco Poeta^1^, Valentina Cioffi^1^, Vittoria Buccigrossi^1^, Merlin Nanayakkara^1^, Melissa Baggieri^2^, Roberto Peltrini^3^, Angela Amoresano^4^, Fabio Magurano^2^ & Alfredo Guarino^1^*

^1^Pediatrics Division, Department of Translational Medical Science, University of Naples Federico II, Naples, Italy. ^2^Department of Infectious Diseases, National Institute of Health, Rome, Italy. ^3^Department of Public Health, University of Naples Federico II, Naples, Italy. ^4^Department of Chemical Sciences, University of Naples Federico II, Naples, Italy.

**
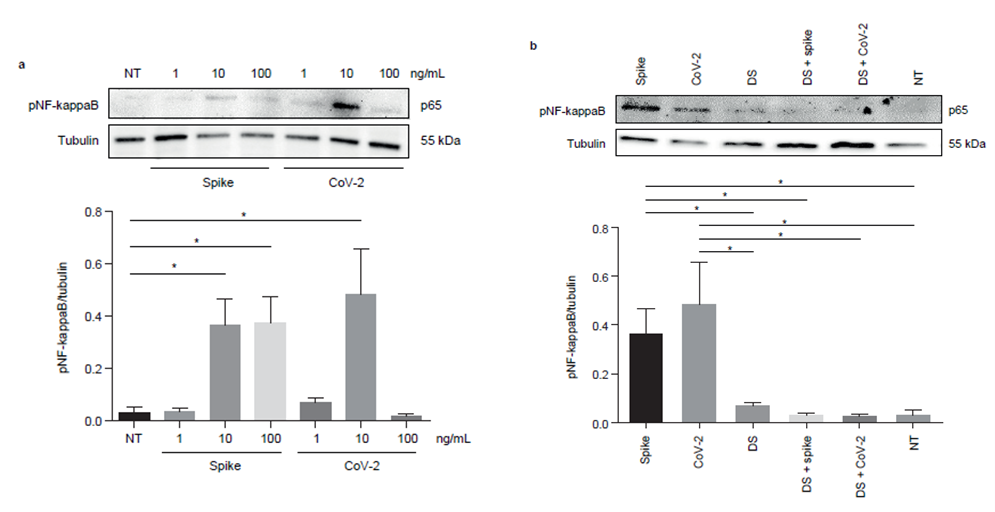
**

**Supplementary Figure 1. NF-kappaB activation and preventive effect of diosmectite (1 h exposure)**

(**a**) Western blot analysis of protein lysates from Caco-2 cells treated for 1 h with different concentrations of spike protein RBD (Spike) and heat-inactivated SARS-CoV-2 (CoV-2) as indicated. (**b**) Western blot analysis of protein lysates from Caco-2 cells treated with spike protein RBD (10 ng/mL) and heat-inactivated CoV-2 (10 ng/mL) for 1 h alone and after pretreatment with diosmectite (DS). The upper line was blotted with anti-pNF-kappaB antibodies and the lower line was blotted with anti-tubulin antibodies as a loading control. A representative image from three independent experiments is shown. The relative levels of pNF-kappaB were normalized to tubulin levels. Bars indicate the means and lines the standard deviations of the three independent experiments. Student’s t‑test. **p* ≤ 0.05. SARS-CoV-2, severe acute respiratory syndrome coronavirus 2; DS, diosmectite; NF-kappaB, nuclear factor kappaB; NT, not treated; p65, primary antibody for NF-kappaB; pNF-kappaB, phosphorylated NF- kappaB. Full-length blots/gels are presented in Supplementary Figure 4.


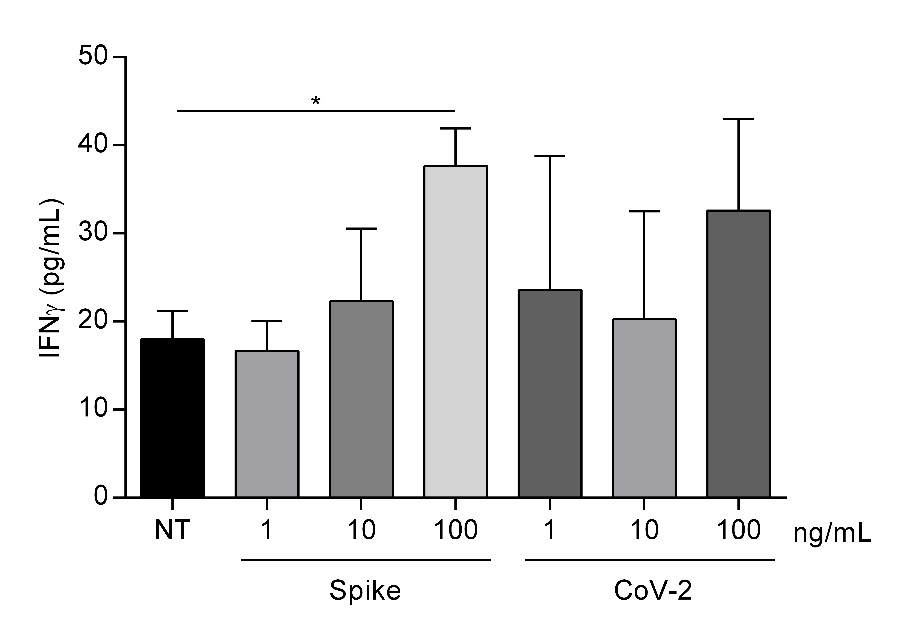


**Supplementary Figure 2. IFN‐γ secretion**

Levels of IFN‐γ measured by ELISA in supernatants of Caco-2 cell cultures treated for 24 h with different concentrations of spike protein RBD (Spike) and heat-inactivated SARS-CoV-2 (CoV-2) as indicated. Bars indicate the means and lines the standard deviations of three independent experiments. Student’s t‑test. **p* ≤ 0.05. ELISA, enzyme-linked immunosorbent assay; IFN‐γ, interferon‐γ; SARS-CoV-2, severe acute respiratory syndrome coronavirus 2; NT, not treated.

**
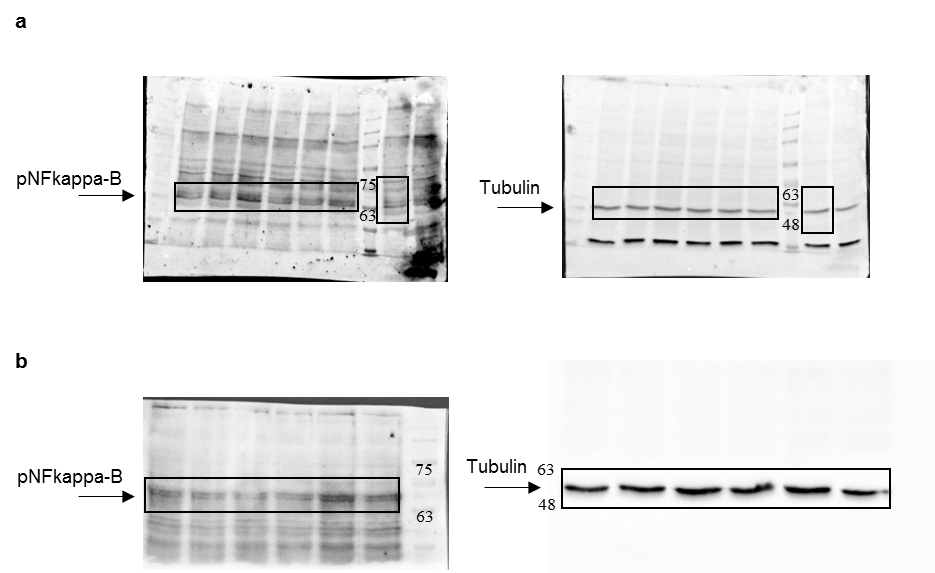
**

**Supplementary Figure 3. Full-length blots/gels of NF-kappaB activation experiments (24 h exposure).**

(**a**) Western blot analysis of protein lysates from Caco-2 cells treated for 24 h with different concentrations of spike protein RBD (Spike) in and heat-inactivated SARS-CoV-2 (CoV-2) as indicated (**Figure 3a)**. (**b**) Western blot analysis of protein lysates from Caco-2 cells treated with spike protein RBD (100 ng/mL) and heat-inactivated CoV-2 (100 ng/mL) for 24 h alone and after pretreatment with diosmectite (DS) (**Figure 3b)**.


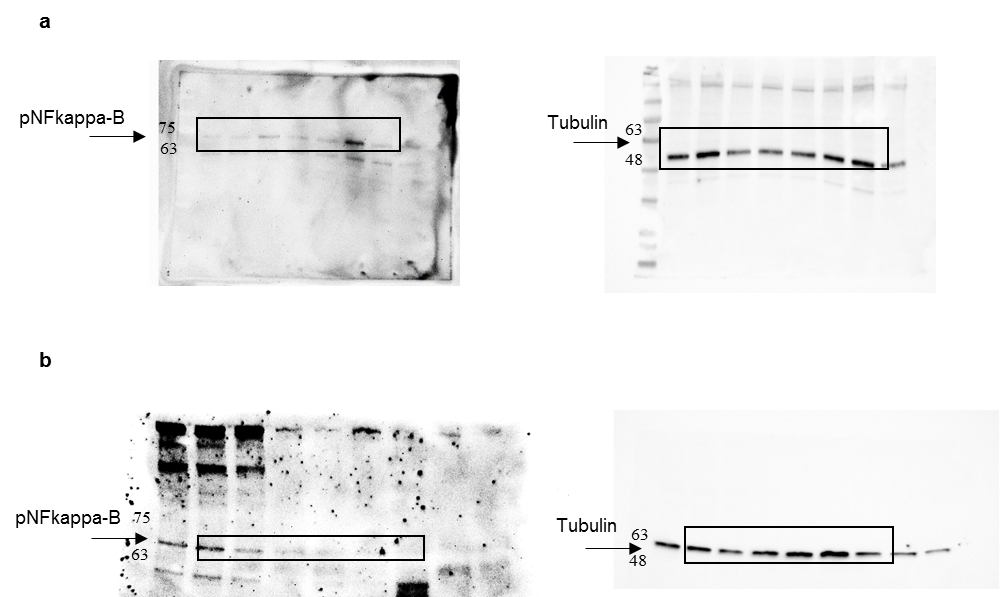


**Supplementary Figure 4. Full-length blots/gels of NF-kappaB activation experiments (1 h exposure)**

(**a**) Western blot analysis of protein lysates from Caco-2 cells treated for 1 h with different concentrations of spike protein RBD (Spike) in and heat-inactivated SARS-CoV-2 (CoV-2) as indicated (**Supplementary** **Figure 1a)**. (**b**) Western blot analysis of protein lysates from Caco-2 cells treated with spike protein RBD (10 ng/mL) and heat-inactivated CoV-2 (10 ng/mL) for 1 h alone and after pretreatment with diosmectite (DS) (**Supplementary Figure 1b)**.
